# *Agrobacterium-*Mediated Genetic Transformation of Taiwanese Isolates of *Lemna aequinoctialis*

**DOI:** 10.3390/plants10081576

**Published:** 2021-07-30

**Authors:** Kuang-Teng Wang, Ming-Chang Hong, Yu-Sheng Wu, Tsung-Meng Wu

**Affiliations:** 1 Department of Aquaculture, National Pingtung University of Science and Technology, Pingtung 91201, Taiwan; P10913002@mail.npust.edu.tw (K.-T.W.); wuys0313@mail.npust.edu.tw (Y.-S.W.); 2 Department and Graduate Institute of Aquaculture, National Kaohsiung University of Science and Technology, Kaohsiung 81157, Taiwan; junkrough.hmc@nkust.edu.tw

**Keywords:** duckweed, transformation, *Agrobacterium tumefaciens*, tissue culture

## Abstract

Duckweed (*Lemna aequinoctialis*) is one of the smallest flowering plants in the world. Due to its high reproduction rate and biomass, duckweeds are used as biofactors and feedstuff additives for livestock. It is also an ideal system for basic biological research and various practical applications. In this study, we attempt to establish a micropropagation technique and *Agrobacterium*-mediated transformation in *L. aequinoctialis*. The plant-growth regulator type and concentration and *Agrobacterium*-mediated transformation were evaluated for their effects on duckweed callus induction, proliferation, regeneration, and gene transformation efficiency. Calli were successfully induced from 100% of explants on Murashige and Skoog (MS) medium containing 25.0 μM 2,4-dichlorophenoxyacetic acid (2,4-D) and 2.0 μM thidiazuron (TDZ). MS medium containing 4.5 μM 2,4-D and 2.0 μM TDZ supported the long-lasting growth of calli. Fronds regenerated from 100% of calli on Schenk and Hildebrandt (SH) medium containing 1.0 μM 6-benzyladenine (6-BA). We also determined that 200 μM acetosyringone in the cocultivation medium for 1 day in the dark was crucial for transformation efficiency (up to 3 ± 1%). Additionally, we propose that both techniques will facilitate efficient high-throughput genetic manipulation in Lemnaceae.

## 1. Introduction

The genus *Lemna* is a member of the Lemnaceae or duckweed family. In nature, there are five genera within this family: *Landoltia*, *Lemna*, *Spirodela*, *Wolffiella,* and *Wolffia* [1]. Duckweed is the smallest and fastest-growing aquatic plant and is widely distributed on the surface of still or slowly flowing water around the world [2,3]. The biomass doubles in 48 h under controlled and axenic conditions, which raises the possibility of using duckweed in bioreactors and feedstock [4,5,6]. In addition, several studies have shown that duckweed has a high starch accumulation rate, low lignin content, and pollution-absorbing ability. It can be used as a reliable material for bioethanol fermentation [7,8,9,10] and could be widely used as an ideal aquatic plant for phytoremediation [11]. Moreover, duckweed is an ideal plant-based system for producing recombinant proteins due to its high protein content. For instance, Biolex Therapeutics produced Locteron^®^, a recombinant IFN-α2b synthesized by a duckweed-based expression system that uses *Lemna minor*, for the large-scale production of recombinant pharmaceuticals and interferons [12].

To date, an *Agrobacterium*-mediated transformation system has been developed for several duckweeds, including *Lemna gibba* [13,14], *Lemna minor* [5,14,15,16,17,18,19,20,21], *Lemna turionifera* [10], *Landoltia punctata* (formerly *Spirodela oligorrhiza*) [22,23], *Spirodela polyrhiza* [5], *Wolffia arrhizal* [24,25], and *Wolffia globosa* [26]. In addition, Liu et al. [27] reported efficient genetic transformation and CRISPR/Cas9-mediated genome editing in *Lemna aequinoctialis* 6002, which is one of the most widely distributed duckweed species in Asia [28,29]. However, the micropropagation and genetic transformation protocol was adopted from that reported in *L. minor* [13,15]. The conditions influencing the virulence and host tissue competence of *Agrobacterium* and consequently the transformation frequencies were not optimized in detail. The plant material of this research was a Taiwanese isolate of *L. aequinoctialis*. It is known that different duckweed isolates might vary in their physiological properties, including hormonal requirements, regeneration potential, and susceptibility to *A. tumefaciens* [15]. Consequently, the protocols of tissue culture and genetic transformation should be revised to the characteristics of duckweed isolates [15].

To facilitate efficient high-throughput genetic manipulation in Lemnaceae, our study focuses on micropropagation and *Agrobacterium*-mediated transformation methodology to better define the optimal basal-medium formulation, plant-growth regulator type and concentration, light, cocultivation time, and acetosyringone (AS) concentration for callus induction, proliferation, regeneration, and genetic transformation in the Taiwanese isolate of *L. aequinoctialis*.

## 2. Results

### 2.1. Callus Induction

**Figure 1:**
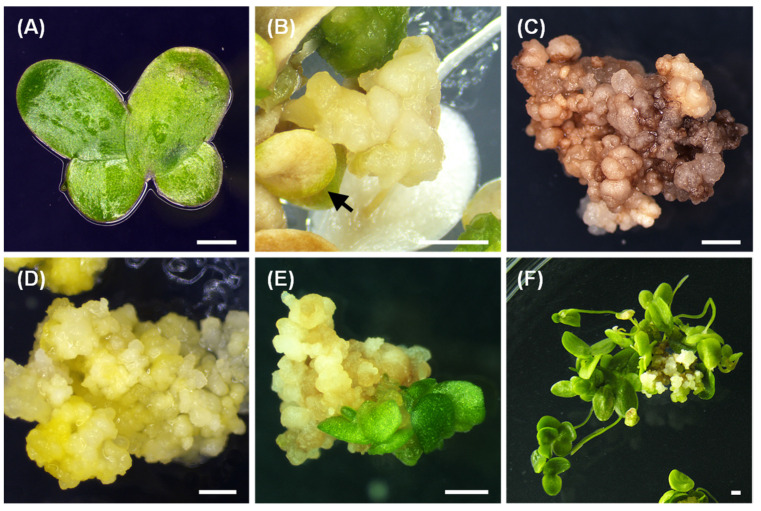
*Lemna aequinoctialis*. (**A**) Fronds of *L. aequinoctialis*; (**B**) fronds were cultured for 8 weeks on MS medium containing 3% sucrose, 25 μM 2,4-D, and 2.0 μM TDZ to induce callus formation. The arrow indicates the senescent frond; (**C**) browning phenomenon of callus cultured on previous CIM; (**D**) callus that was separated from the bleached frond explant and grown for an additional 4 weeks on MS medium containing 3% sucrose, 4.5 μM 2,4-D, and 2.0 μM TDZ for callus proliferation; (**E**,**F**) plants regenerating from callus incubated for 2 (**E**) and 4 (**F**) weeks on SH medium containing 1% sucrose and 1.0 μM 6-BA. Bar = 1.0 mm.

Two to three-week-old fronds of *Lemna aequinoctialis* (Figure 1A) were used to investigate the effects of various combinations of plant-growth regulators (PGRs) on callus induction. The types and concentrations of PGRs had a significant (*p* < 0.05) effect on callus induction (Table 1). Frond proliferation was significantly retarded during the first 2 weeks, fronds started to curl irregularly in all treatments, and white or yellow senescent fronds were observed after 4 weeks of cultivation. Callus induction was slow and first visible after 8 weeks in culture. The callus arose most often from the meristematic region on the ventral surface of the frond. After 10 weeks in culture, different types of callus were obtained. The calli on media supplemented solely with 2,4-D were friable, nodular and whitish to light yellowish-colored (Figure S1A). The calli developed on media containing 2,4-D combined with cytokinins were also friable and nodular, but yellowish and greenish-colored (Figure S1B). Calli could be induced in each treatment, whereas 2,4-D combined with both cytokinins could significantly increase the induction rate, especially with the addition of TDZ. The most successful callus induction was observed on callus-induction medium (CIM) with 25.0 µM 2,4-D combined with TDZ (0.5 or 2.0 µM) or 6-BA (2.0 µM), where 100% of explants produced calli. However, more calli induced from 25.0 µM 2,4-D/2.0 µM TDZ showed a friable hard texture and golden yellow morphology (Figure 1B). The calli that were subcultured in the same medium showed retarded growth and browning phenomena (Figure 1C). Thus, the callus-proliferation medium was determined. In summary, the most effective CIM was MS medium containing 3% (*w*/*v*) sucrose, 0.3% (*w*/*v*) Phytagel, and 2,4-D (25.0 μM) combined with TDZ (2.0 μM) at pH 5.7.

### 2.2. Nodular Callus Proliferation

**Figure 2:**
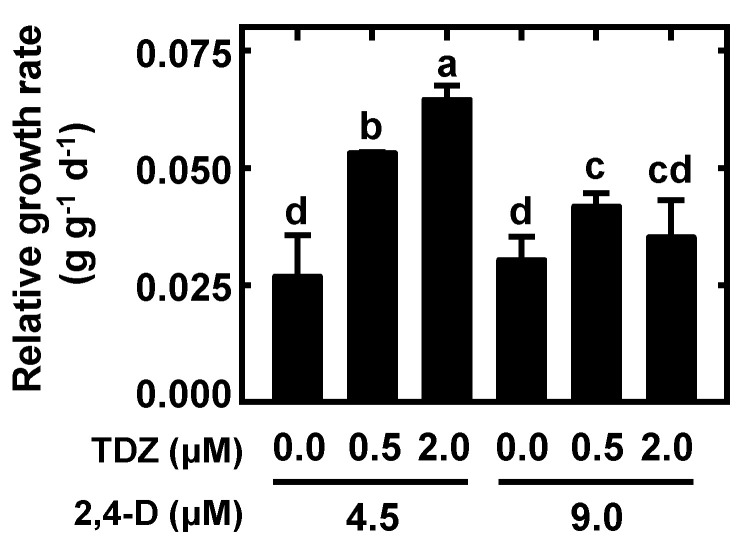
The effects of 2,4-D and TDZ on callus proliferation. Calli were cultured on the MS medium containing 3% sucrose and different plant growth regulators. Data (mean ± SD) with different letters among treatments significantly differ (*p* < 0.05).

Because callus proliferation was very slow on CIM, six combinations of 2,4-D with TDZ were tested to determine if they would enhance the rate of callus growth. Nodular callus-proliferation medium (NCPM) with 4.5 µM 2,4-D and 2.0 µM TDZ supported the significant growth rate of calli (Figure 2), which prevented growth cessation and browning. The morphology of calli derived from the effective NCPM was golden yellow and friable with a hard texture and consisted of small, connected amorphous masses (Figure 1D). Furthermore, the abovementioned medium supported callus proliferation for over 6 months by subculturing periodically. Additionally, the most effective NCPM was MS medium containing 3% (*w*/*v*) sucrose, 0.3% (*w*/*v*) Phytagel, and 2,4-D (4.5 μM) combined with TDZ (2.0 μM) at pH 5.7.

### 2.3. Frond Regeneration

Both SH and MS basal media supplemented with 6-BA were evaluated for their ability to regenerate fronds using nodular calli derived from the most effective NCPM (Table 2). The results showed that SH medium was superior to MS medium independent of cytokinin addition, whereas a 60 ± 5% regeneration rate was obtained in SH medium without the addition of 6-BA. In contrast, a ½ MS and MS medium poorly supported frond regeneration (0%). The formation of frond-like clusters and green structures was observed within 2–3 weeks after transferring nodular calli from NCPM to frond regeneration medium (FRM) (Figure 1E). The highest frond regeneration rate (100%) and number of fronds regenerated per callus (42 ± 4) was observed on FRM composed of SH medium containing 1.0 µM 6-BA after 4 weeks of cultivation. An example of *L. aequinoctialis* regenerating from callus tissue is shown in Figure 1F. Eventually, the most effective FRM was SH medium containing 1.0% (*w*/*v*) sucrose, 0.3% (*w*/*v*) Phytagel, and 6-BA (1.0 μM) at pH 5.7.

### 2.4. Optimization of the Transformation Protocol

**Figure 3:**
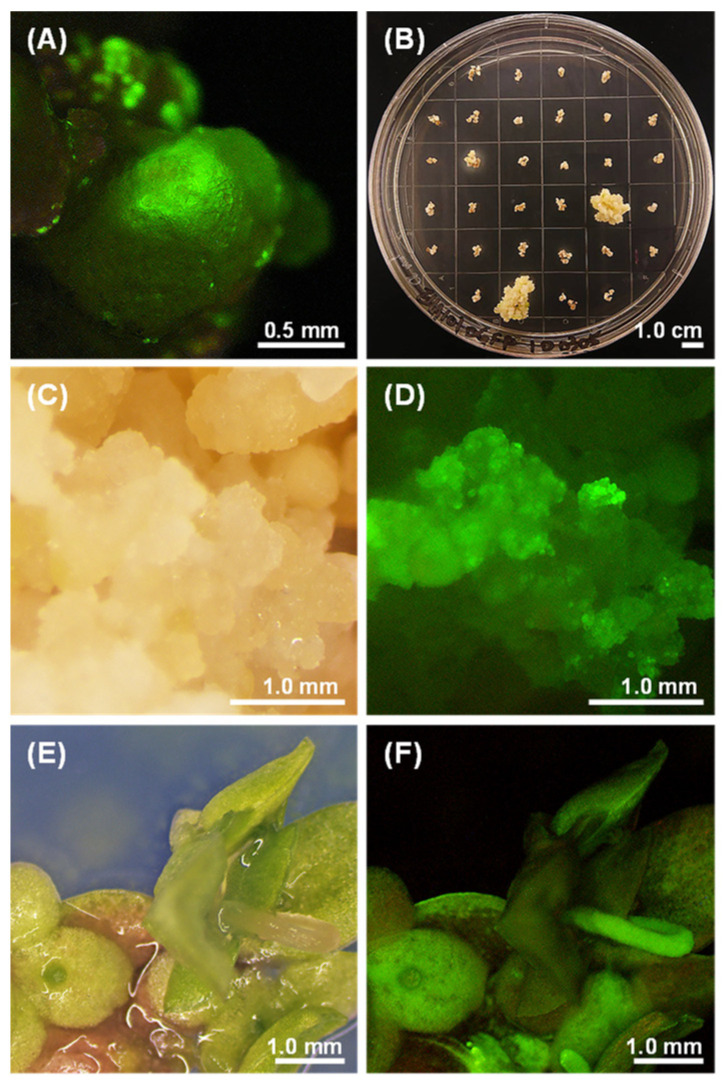
Stable transformation and regeneration of *L. aequinoctialis* callus with a GFP expression construct. (**A**) Transformed nodular calli showed GFP expression under selection medium at day 9; (**B**) newly divided calli were observed after 10 weeks of selection; (**C**) newly divided calli were observed under bright-field and (**D**) GFP channel with long-pass filter; (**E**) transformed fronds were observed under bright-field and (**F**) GFP channel with long-pass filter.

To develop an efficient procedure for the stable transformation of *Lemna aequinoctialis*, we elucidated the effect of altering the concentrations of acetosyringone (AS), coculture days, and photoperiods on transformation efficiency (Table 3). We monitored GFP expression in nodules throughout the selection process after transferring the cocultured calli to the selection medium (NCPM-CH), which contained 50 mg L^−1^ hygromycin and 275 mg L^−1^ cefotaxime. The results showed that the maximum transformation frequency was observed in nodular calli cocultivated with the pD-clone1-harboring *Agrobacterium* in NCPM containing 200 µM AS in the dark for 1 day. During the early stage of selection, many GFP-expressing calli (Figure 3A) were observed, and a 49 ± 4% transformation frequency was obtained under NCPM-CH on day 9. In addition, we observed GFP-positive cells on many nodules that appeared dead following the selection process. After 10 weeks of selection, the newly divided calli showed GFP expression (Figure 3B–D). Furthermore, relatively few stable transformants (3 ± 1%) were recovered compared with the large number of GFP-positive cells at early stages. These observations indicated that only a small fraction of cells transiently expressing GFP became stably transformed. Subsequently, we transferred the resistant calli into FRM containing 50 mg L^−1^ hygromycin for frond regeneration. Regenerated fronds with GFP expression could be found at a 100% rate of regeneration after 4 weeks of regeneration. Finally, the regenerated fronds were transferred to SH medium and subcultured periodically. Transformants that had been propagated vegetatively for up to 1 year exhibited a uniform GFP signal, indicating the long-term stability of GFP expression (Figure 3E,F). Accordingly, the optimal transformation condition for *L. aequinoctialis* was observed in nodular calli cocultured in NCPM containing 200 µM AS for 1 day in the dark.

### 2.5. Molecular Analysis of Putative Transformants

**Figure 4:**
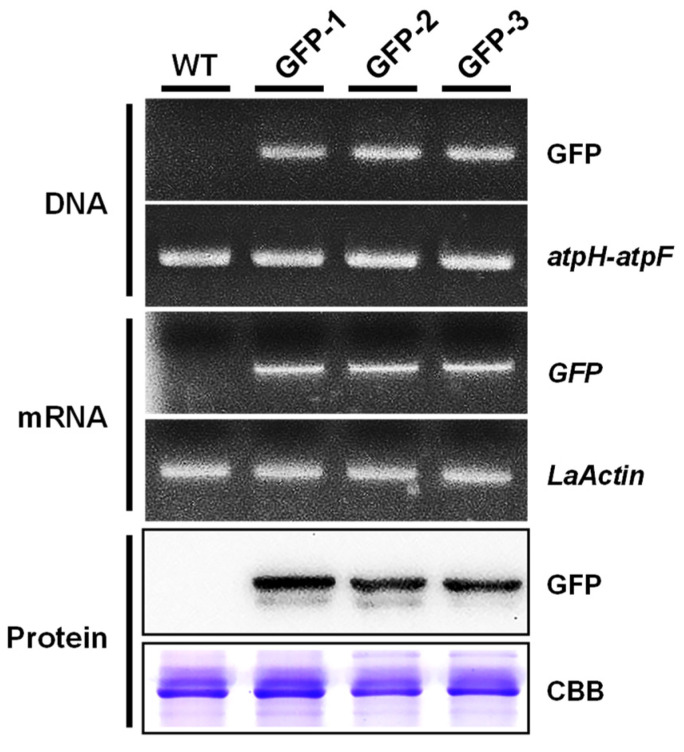
Molecular analysis of putative transformants from DNA, mRNA, and protein. The *atpF-atpH* marker, *LaActin*, and coomassie brilliant blue (CBB) staining were used as internal controls for each molecular analysis, respectively.

To verify the putative transformants with the stable integration of the transgene, DNA, mRNA, and protein levels of the transgene (GFP) were evaluated in the three transgenic lines with the strongest GFP fluorescence (Figure 4). First, we verified the incorporation of the transgene using PCR-based analysis. According to the genomic PCR analysis, wild type and transformants showed *atpH*-*atpF* fragments, but GFP signals were only detected in the three transgenic lines. The positive RT-PCR results indicated that GFP transcripts were also detected in the three transgenic lines. Western blot analysis revealed the presence of a ~27-kDa band corresponding to the GFP protein in the three transgenic lines. Overall, the transformants were confirmed by the molecular analytical results above.

## 3. Discussion

Previous studies have implemented callus induction on species within Lemnoideae and have revealed that both the type and concentration of PGRs can significantly enhance the callus induction rate [25,30,31]. A crucial role is played by 2,4-D in callus induction in many plant species [32,33,34]. In 1997, Moon and Stomp [35] reported that MS medium supplemented with 20.0 or 50.0 µM 2,4-D alone could induce callus formation in *Lemna gibba*, but only ~10% of fronds produced calli. In this study, we found that *L. aequinoctialis* calli could be induced on MS medium containing 25.0 µM 2,4-D and 2.0 µM TDZ at a rate of 100%, which was better than the results from Liu et al. [27]. Our results showed that the optimal balance between auxin and cytokinin could significantly enhance the callus-induction rate. Several studies have demonstrated similar results in other duckweeds. For example, Chhabra et al. [15] found that B5S1.0 medium containing 50.0 µM 2,4-D and 5.0 µM TDZ could induce hard nodular calli in *L. minor*. In addition, Huang et al. [30] reported that the optimum medium for callus induction was MS basal medium supplemented with 15 mg L^−1^ 2,4-D (~67.9 µM) and 2 mg L^−1^ 6-BA (~9.01 µM) for *Landoltia punctata*; Yang et al. [5] reported that the optimum medium for callus induction was half-strength MS basal medium containing 22.62 µM 2,4-D and 8.88 µM 6-BA in *Spirodela polyrhiza*; and Khvatkov et al. [26] reported that the optimum medium for callus induction was SH medium containing 5.0 mg L^−1^ 2,4-D (~22.6 µM) and 0.5 mg L^−1^ 6-BA (~2.25 µM) in *Wolffia arrhiza*.

Our results showed that nodular callus-production medium (NCPM) with 4.5 μM 2,4-D and 2.0 μM TDZ could significantly improve callus proliferation. For *L. minor*, Cantó-Pastor et al. [13] transferred calli to culture medium containing 1.0 μM 2,4-D and 2.0 μM 6-BA, which had lower 2,4-D concentrations than the callus-induction medium. Firsov et al. [16] transferred calli to culture medium with 2.0 mg L^−1^ 2,4-D (~9.04 μM) for subsequent growth and proliferation in *L. gibba*. Therefore, we found that FRM with 1.0 μM 6-BA in SH medium had a 100% rate of regeneration. For *L. gibba*, a 100% plant regeneration rate was observed on FRM with 1.0 μM 6-BA in SH medium [36]. For *Spirodela punctata*, a 95–98% plant regeneration rate was attained on FRM with 1.0 mg L^−1^ 2ip in McCown Woody Plant medium [37]. It has been previously reported that *L. gibba* G3 and *W. arrhiza* fronds could also regenerate on PGR-free SH medium [25,35,36]. This indicates that SH medium is crucial for duckweed frond regeneration, which could be promoted by cytokinin addition. Above all, the selection and concentration of hormones could affect the formation of callus proliferation and frond regeneration in different duckweed species.

Here, by establishing the transformation protocol, we achieved a 3 ± 1% stable transformation success rate for *Agrobacterium*-mediated transformation in *L. aequinoctialis*. In addition, the stable transformation efficiency obtained was approximately 3.8% [15] and 4% [20] in *L. minor*, a different species of the same genus, or 0.5–5% in *Spirodela oligorrhiza* [23] and 0.4% in *W. arrhizal* [24,38]. Thus, we developed a protocol that obtained a similarly stable transformation efficiency that is feasible for *Agrobacterium*-mediated transformation in *L. aequinoctialis*. Acetosyringone (AS) has previously been shown to aid in the transformation of several crops, such as cotton, rice, and wheat [23]. Researchers have also found that adding AS at a low pH to cocultivation medium was effective in enhancing T-DNA transfer. For example, Cantó-Pastor et al. [13] used 200 µM AS during the *Agrobacterium* transformation of *L. minor*. In addition, Vunsh et al. [23] supplemented *S. oligorrhiza* with 100 µM AS at pH 5.6. These conditions had similar results to those found in the transformation protocol of *S. oligorrhiza*, *L. gibba* and *L. minor* [13,14,15]. Our study showed that the addition of 200 µM AS to the cocultivation medium at pH 5.2 could result in the maximum transformation rate. The cocultivation period and photoperiod were also important factors that could affect the transformation efficiency, while shorter or longer cocultivation periods led to unsuccessful transformation [39]. In our study, we determined 1 day in the dark to be the optimal duration for the cocultivation of *L. aequinoctialis* and *Agrobacterium*. Vunsh et al. [23] and Yang et al. [21] showed the same cocultivation conditions for *S. oligorrhiza* and *S. polyrhiza*. We know that longer cocultivation periods of approximately 4–5 days could cause the over-proliferation of *Agrobacterium* around the plant materials, ultimately leading to the death of the plant tissue [26]. Interestingly, Chhabra et al. [15] showed that maximum transformation frequencies were observed in nodular calli of *L. minor* cocultured for 3 days under a 16 h light/8 h dark photoperiod. They suggested that the possible reason for the difference was due to different physiological properties among duckweed species. Eventually, we successfully regenerated fronds from the transgenic calli. These fronds stably expressed green fluorescence through vegetative proliferation for several generations (up to 1 year). To date, transgenic fronds have shown uniform GFP activity, which suggests their ability to stably inherit transgenes through mitotic divisions.

However, several factors greatly affect the transformation efficiency, including plant species, type of explants, *A. tumefaciens* strain, binary vector types, infection methods, surfactant addition, and so on [39]. In several previous studies, such as on banana [40], chickpea [41], and cowpea [42], it has been shown that the combination of vacuum infiltration and sonication can greatly improve the efficiency of *Agrobacterium* infection. In comparison to previous studies of *Agrobacterium*-mediated transformation in duckweeds [13,14,15,16,17], Liu et al. [27] also suggested that the increased transformation efficiency likely resulted from the use of sonication coupled with vacuum infiltration infection. We believe the two strategies can be used to enhance the transformation efficiency in Taiwanese isolates of *L. aequinoctialis* in the future.

## 4. Materials and Methods

### 4.1. Plant Material and Growth Conditions

*Lemna aequinoctialis* was collected from a pool at National Pingtung University of Science and Technology (NPUST), Pingtung, Taiwan. The duckweed species was identified by an *atpF*-*atpH* noncoding spacer [43]. After sterilization using 0.6% NaHClO for 2 to 3 min and washing 6 to 8 times with sterilized water, the plants were cultured in 90 × 20 mm Perti dishes containing 50 mL Schenk and Hildebrandt (SH) medium with 1% sucrose and 0.30% (*w*/*v*) Phytagel (Sigma, St. Louis, MO, USA) at pH 5.7. All cultures were maintained at 27 ± 1 °C under a 12 h photoperiod of cool-white fluorescent light of 80 μmol photons m^−2^ s^−1^ intensity. An axenic clone was produced and maintained in our laboratory for 4 years. Two to three-week-old axenic fronds were used for callus induction.

### 4.2. Callus Induction

To evaluate the optimal conditions for callus induction, fronds (~3.5 mm in length) were grown on MS medium containing 3% (*w*/*v*) sucrose and 0.3% (*w*/*v*) Phytagel at pH 5.7. Different combinations of plant-growth regulators were added in the medium. For cytokinins, we used either 6-benzyladenine (6-BA) or thidiazuron (TDZ) at 0, 0.5, 1 and 2 μM; for auxins, we used 2,4-dichlorophenoxyacetic acid (2,4-D) at 5, 10, 25 and 50 μM. Thirty-two fronds were cultured in sterilized 150 × 20 mm Petri dishes containing 100 mL solid medium for callus induction. The explants were maintained under a 12/12 h photoperiod at 27 ± 1 °C (80 μmol photons m^−2^ s^−1^). Each treatment was performed in triplicate. The efficiency of callus induction on each plate was calculated after 10 weeks of cultivation.

### 4.3. Nodular Callus Proliferation

To evaluate the optimal conditions for callus proliferation, calli (~5 mm in diameter) derived from the most effective callus-induction medium (CIM) were cultured on MS medium containing 3% (*w*/*v*) sucrose, 0.3% (*w*/*v*) Phytagel, and either 2,4-D at 4.5 or 9.0 μM combined with TDZ (0, 0.5 and 2 μM) at pH 5.7. Five calli were cultured in sterilized 90 × 20 mm Petri dishes containing 50 mL solid medium under a 12/12 h photoperiod at 27 ± 1 °C (80 μmol photons/m^2^/s). Each treatment was performed in triplicate. The relative growth rate (RGR) of calli during 4 weeks of cultivation was calculated with the following equation [44]: RGR = (lnW_t_ − lnW_0_)/(t_t_ − t_0_), where W_t_ is the fresh weight at time t_t_ and W_0_ is the initial fresh weight at the beginning of the treatment. The nodular calli derived from the most effective nodular callus-proliferation medium (NCPM) were then used for frond regeneration and gene transformation.

### 4.4. Frond Regeneration

To determine the optimal conditions for frond regeneration, calli (~5 mm in diameter) derived from the most effective NCPM were cultured on both MS and SH media containing 3% and 1% (*w*/*v*) sucrose, respectively, 0.3% (*w*/*v*) Phytagel, and 6-BA (0, 1, 2 μM) at pH 5.7. Five calli were cultured in sterilized 90 × 20 mm Petri dishes containing 50 mL solid medium under a 12/12 h photoperiod at 27 ± 1 °C (80 μmol photons m^−2^ s^−1^). Each treatment was performed in triplicate. The efficiency of frond regeneration on each plate was calculated after 4 weeks of cultivation.

### 4.5. Agrobacterium Strain and Binary Vector

The green fluorescence protein (GFP) expression vector pD-clone1 [45] was introduced into *Agrobacterium* strain EHA101 and used for genetic transformation. pD-clone1 contained two gene expression cassettes, including the *GFP* protein with the hygromycin selection marker (hygromycin phosphotransferase gene, *Hpt*). *GFP* was driven by the maize *ubiquitin1* promoter together with its intron (*Ubi1*) promoter and terminated by the *Nos* poly (A) signal.

### 4.6. Optimization of the Transformation Protocol

To develop a genetic transformation protocol for *Lemna aequinoctialis*, various parameters influencing transformation efficiency were evaluated. Three days prior to the transformation experiment, *Agrobacterium tumefaciens* (pD-clone1) from a freshly streaked plate was inoculated in 3 mL of liquid YEP medium (yeast extract 1.0 g L^−1^, bactopeptone 10.0 g L^−1^, NaCl 5 g L^−1^) containing ampicillin (100 mg L^−1^) and kanamycin (50 mg L^−1^) and grown overnight on a rotary shaker (180 rpm) at 27 °C. The next day, 0.5 mL of this primary culture was inoculated in 50 mL subculture medium (3.0 g L^−1^ K_2_HPO_4_, 1.0 g L^−1^ NaH_2_PO_4_, 1.0 g L^−1^ NH4Cl, 0.3 g L^−1^ MgSO_4_·7H_2_O, 0.15 g L^−1^ KCl, 0.01 g L^−1^ CaCl_2_, 2.5 mg L^−1^ FeSO_4_·7H_2_O, and 5 g L^−1^ glucose) containing ampicillin (100 mg L^−1^) and kanamycin (50 mg L^−1^). The culture was grown at 27 °C with shaking (180 rpm) until the OD_600_ reached 0.6–0.8. The bacteria were harvested by centrifuging at 5000 rpm for 7 min at 4 °C and resuspended in 10 mL MS liquid medium. After soaking for 2 min, the calli (~100 calli/treatment) were blotted dry on sterile filter paper and cocultivated on the most effective NCPM (pH 5.2) supplemented with 0, 50, 100, and 200 µM acetosyringone (AS) for 1–3 days at 27 ± 1 °C under a 12/12 h photoperiod or dark conditions.

After cocultivation, nodular calli from each treatment were collected and washed with sterile distilled water at least 20 times, followed by washing with MS liquid medium containing 275 mg L^−1^ cefotaxime once per hour for five hours on a shaker at 110 rpm. The mixture was left to culture overnight. After washing, the calli were blotted dry and cultured on selection medium (the most effective NCPM containing 50 mg L^−1^ hygromycin and 275 mg L^−1^ cefotaxime) (NCPM-CH) under a 12/12 h photoperiod at 27 ± 1 °C (80 μmol photons/m^2^/s).

After culturing on the selection medium for 3 days, we started to screen calli showing GFP signals under a fluorescence stereomicroscope (SteREO Discovery. V8, Carl Zeiss Microscopy, Pleasanton, CA, USA) until the nodular calli began to proliferate, which were regarded as putative transformants. The frequency of GFP expression was calculated as the number of calli showing GFP out of the total number of treated cells. Hygromycin-resistant calli could be found at approximately 10 weeks. The putative transformants were transferred to regeneration medium containing 50 mg L^−1^ hygromycin for frond regeneration.

### 4.7. Genomic PCR Analysis of Transgene Integration

Genomic DNA was extracted from wild-type and putative transformants by using an RBC Real Genomics Genomic DNA Extraction Kit (YGP50, RBCBioscience) and used as a template for PCR analysis to test the presence for *GFP* using the following specific primers: GFP-F: 5′-CAAGGGCGAGGAGCTGTT-3′; and GFP-R: 5′-CTTGTACAGCTCGTCCATGC-3′. The chloroplast marker *atpF*-*atpH* was amplified as an endogenous control [43]. The PCR program was as follows: 94 °C denaturation for 10 min, followed by 30 cycles at 94 °C for 30 s, 50 °C for 30 s, and 72 °C for 40 s, and a final extension at 72 °C for 7 min. The PCR products were resolved by electrophoresis in 1.2 % (*w*/*v*) agarose gel and stained with SafeView™ Classic (Applied Biological Materials Inc., Richmond, BC, Canada).

### 4.8. RT- PCR Analysis of Transgenic Duckweed

Total RNA was isolated from wild-type and putative transformants using TRIzol reagent (Invitrogen, CA, USA), and cDNA synthesis involved the use of an MMLV reverse transcriptase first-strand cDNA synthesis kit (Epicentre Biotechnologies, Madison, WI, USA). cDNAs were used as a template for RT-PCR analysis to determine *GFP* and *Lemna aequinoctialis Actin* (*LaActin*, GenBank accession no. MZ394729) expression using the following specific primers: the same primer pairs of GFP; LaActin-F: 5′-GACGCAGATCATGTTCGAGA-3′; and LaActin-R: 5′-TGGTTCCACCACTAAGCACA-3′. PCR amplification was performed as mentioned above. Three batches of independently isolated total RNA samples underwent three rounds of RT-PCR. All tests were repeated at least three times, and the results from one repeat are shown in the figures. *LaActin* was used as the internal control.

### 4.9. Western Blot Analysis of Transgenic Duckweed

Total protein extracted from the fronds of wild-type plants and putative transformants (15 μg each lane) was subjected to SDS-PAGE using a 12% polyacrylamide gel and Western blot analysis with a monoclonal antibody against GFP (sc-9996, Santa Cruz Biotechnology, Inc., Dallas, TX, USA) as described in [46]. Immunoreactivity was detected by the enhanced chemiluminescence method (Amersham International).

### 4.10. Statistical Analysis

A one-way analysis of variance (ANOVA) was used to analyze the data. When the ANOVA identified differences among the groups, Duncan’s multiple range test was conducted to examine significant differences among the treatments in SAS software (SAS Institute, Cary, NC, USA). The level of significance was *p* < 0.05.

## 5. Conclusions

In conclusion, we developed an efficient micropropagation technique and achieved stable transformation in *Lemna aequinoctialis* (Figure S2). Our results showed that a 100% callus induction rate was observed under CIM with 25.0 µM 2,4-D and 2 µM TDZ, the best callus proliferation was observed under NCPM with 4.5 μM 2,4-D and 2.0 μM TDZ, and a 100% frond regeneration rate was observed under frond regeneration medium (FRM) with 1 μM 6-BA. We achieved the stable transformation of calli derived from the optimal NCPM using *Agrobacterium tumefaciens* strain EHA101 harboring pD-clone1. A stable transformation frequency (3.13 ± 1.08%) was observed in calli cocultivated with *Agrobacterium* on NCPM containing 200 μM AS for 1 day in the dark. Therefore, the protocol for micropropagation and *Agrobacterium*-mediated transformation could be a good foundation for the transgenic engineering of *L. aequinoctialis*.

## Figures and Tables

**Table 1:** The effects of the type and concentration of plant growth regulators on callus induction from fronds after 10 weeks in culture.

2,4-D ^1^ (μM)		Cytokinin ^2^ (μM)	% Fronds Forming Callus ^3^
5		0.0	57 ± 6 ^gf^
10			65 ± 5 ^ef^
25			50 ± 9 ^g^
50			27 ± 6 ^h^
5	6-BA	0.5	28 ± 14 ^h^
		1.0	55 ± 5 ^gf^
		2.0	55 ± 5 ^gf^
10		0.5	65 ± 5 ^ef^
		1.0	67 ± 3 ^ef^
		2.0	73 ± 14 ^e^
25		0.5	87 ± 3 ^bcd^
		1.0	95 ± 5 ^abc^
		2.0	100 ± 0 ^a^
50		0.5	72 ± 3 ^e^
		1.0	93 ± 6 ^abc^
		2.0	87 ± 6 ^bcd^
5	TDZ	0.5	75 ± 5 ^de^
		1.0	88 ± 3 ^bc^
		2.0	85 ± 5 ^cd^
10		0.5	93 ± 6 ^abc^
		1.0	95 ± 5 ^abc^
		2.0	87 ± 12 ^bcd^
25		0.5	100 ± 0 ^a^
		1.0	95 ± 5 ^abc^
		2.0	100 ± 0 ^a^
50		0.5	92 ± 10 ^abc^
		1.0	98 ± 3 ^ab^
		2.0	97 ± 3 ^abc^

^1^ 2,4-D, 2,4-dichlorophenoxyacetic acid. ^2^ TDZ, thidiazuron; 6-BA, 6-benzylaminopurine. ^3^ Data (mean ± SD) with different letters among treatments significantly differ (*p* < 0.05).

**Table 2:** The effects of basal medium type and strength and 6-BA concentration on the frequency and efficiency of frond regeneration from callus after 4 weeks in culture.

Basal Medium ^1^	6-BA (μM)	% Callus Regenerating Fronds ^2^	Number of Fronds Regenerated/Callus Piece ^2^
	0	0 ^d^	0 ^D^
½SH	1	0 ^d^	0 ^D^
	2	61 ± 5 ^b^	9 ± 2 ^C^
	0	60 ± 5 ^b^	10 ± 2 ^C^
SH	1	100 ± 0 ^a^	42 ± 4 ^A^
	2	47 ± 2 ^c^	18 ± 3 ^B^
	0	0 ^d^	0 ^D^
½MS	1	0 ^d^	0 ^D^
	2	0 ^d^	0 ^D^
	0	0 ^d^	0 ^D^
MS	1	0 ^d^	0 ^D^
	2	0 ^d^	0 ^D^

^1^ SH, Schenk and Hildebrandt (1972); MS, Murashige and Skoog (1962); ½, half-strength. ^2^ Data (mean ± SD) within each column followed by different letters are significantly different (*p* < 0.05).

**Table 3:** The effects of different transformation parameters on GFP expression in nodular calli of *L. aequinoctialis* cocultivated with *A. tumefaciens* strain EHA 101 harboring binary vector pD-clone1.

AS ^1^ (µM)	Coculture (Day)	Photoperiod	GFP Expression ^2^ (%)	
			Day 9	Week 10
0	1	12 h light/12 h dark	0 ^e^	0 ^B^
		24 h dark	0 ^e^	0 ^B^
	2	12 h light/12 h dark	0 ^e^	0 ^B^
		24 h dark	0 ^e^	0 ^B^
	3	12 h light/12 h dark	0 ^e^	0 ^B^
		24 h dark	0 ^e^	0 ^B^
50	1	12 h light/12 h dark	0 ^e^	0 ^B^
		24 h dark	0 ^e^	0 ^B^
	2	12 h light/12 h dark	0 ^e^	0 ^B^
		24 h dark	0 ^e^	0 ^B^
	3	12 h light/12 h dark	0 ^e^	0 ^B^
		24 h dark	0 ^e^	0 ^B^
100	1	12 h light/12 h dark	0 ^e^	0 ^B^
		24 h dark	3 ± 3 ^de^	0 ^B^
	2	12 h light/12 h dark	0 ^e^	0 ^B^
		24 h dark	0 ^e^	0 ^B^
	3	12 h light/12 h dark	0 ^e^	0 ^B^
		24 h dark	0 ^e^	0 ^B^
200	1	12 h light/12 h dark	2 ± 2 ^de^	0 ^B^
		24 h dark	49 ± 4 ^a^	3 ± 1 ^A^
	2	12 h light/12 h dark	2 ± 2 ^de^	0 ^B^
		24 h dark	14 ± 2 ^c^	0 ^B^
	3	12 h light/12 h dark	4 ± 2 ^d^	0 ^B^
		24 h dark	25 ± 5 ^b^	0 ^B^

^1^ AS, acetosyringone. ^2^ Data (mean ± SD) within each column followed by different letters are significantly different (*p* < 0.05).

## Data Availability

The authors confirm that the data supporting the findings of this study are available within the article.

## Supplementary Materials

The following are available online at https://www.mdpi.com/article/10.3390/plants10081576/s1, Figure S1: The callus appearances of *Lemna aequinoctialis*, Figure S2: The scheme for *Agrobacterium*-mediated genetic transformation of Taiwanese isolates of *Lemna aequinoctialis*.
