# Supplementary material for: Agrobacterium-Mediated Genetic Transformation of Taiwanese Isolates of Lemna aequinoctialis
Source: Plants (Basel). 2021 Jul 30;10(8):1576. doi: 10.3390/plants10081576 (PMC8401387; doi:10.3390/plants10081576)
Supplement: Supplementary file 1 [file plants-10-01576-s001.zip › Figure S1.pdf]

# Figure S1

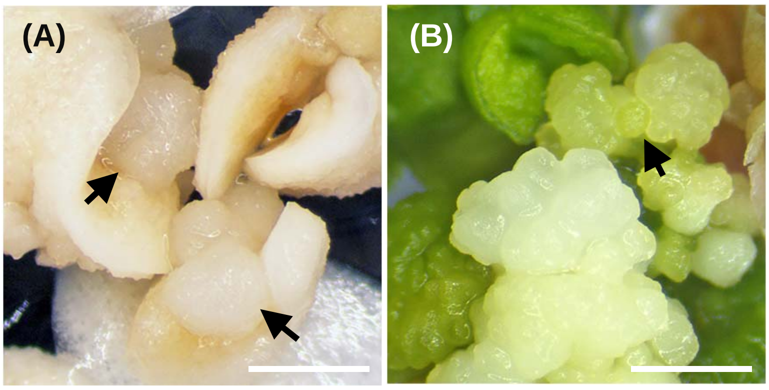


Figure S1. The callus appearances of *Lemna aequinoctialis*. (A) Fronds were cultured for 10 weeks on CIM medium supplemented solely with 25 μM 2,4-D to induce callus formation. The arrows indicated the whitish to light yellowish callus; (B) Fronds were cultured for 10 weeks on CIM medium supplemented with 25 μM 2,4-D and 2.0 μM 6-BA to induce callus formation. The arrow indicated the greenish callus. Bar = 1.0 mm.
