# Supplementary material for: Agrobacterium-Mediated Genetic Transformation of Taiwanese Isolates of Lemna aequinoctialis
Source: Plants (Basel). 2021 Jul 30;10(8):1576. doi: 10.3390/plants10081576 (PMC8401387; doi:10.3390/plants10081576)
Supplement: Supplementary file 1 [file plants-10-01576-s001.zip › Figure S2.pdf]

Figure S2

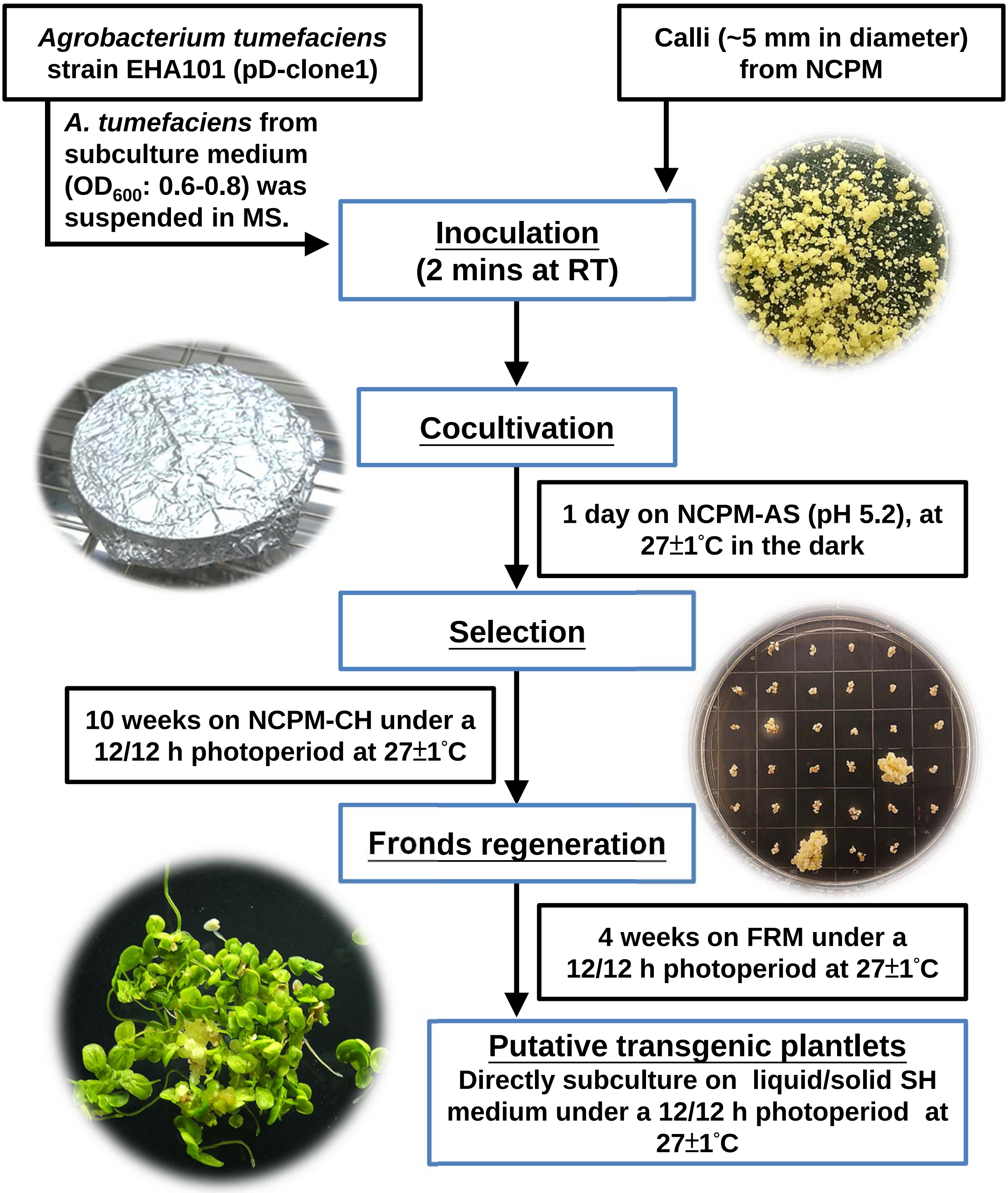

Figure S2. The scheme for *Agrobacterium*-mediated genetic transformation of Taiwanese isolates of *Lemna aequinoctialis*.
